# Editorial: Interdisciplinary approaches to address health disparities for intellectual and developmental disabilities from underserved communities

**DOI:** 10.3389/fped.2025.1745879

**Published:** 2025-12-05

**Authors:** Eric J. Moody, Brian Barger, Nuri M. Reyes, Jiwon Lee

**Affiliations:** 1 Wyoming Institute for Disabilities, College of Health Sciences, University of Wyoming, Laramie, WY, United States; 2 School of Public Health, Population Health Sciences, Center for Leadership in Disability, Georgia State University, Atlanta, GA, United States; 3 Department of Pediatrics, School of Medicine, University of Colorado, Anschutz Medical Campus, Aurora, CO, United States; 4 School of Nursing, Byrdine F. Lewis College of Nursing and Health Professions, Georgia State University, Atlanta, GA, United States

**Keywords:** intellectual and development disabilities, person-centered, interdisciplinary, underserved, global perspective

Individuals with intellectual and developmental disabilities (IDD) account for a significant proportion of the population with about 18% having any type of developmental disability and about 1% with intellectual disabilities globally (1, 2). Prevalence in low- and middle-income countries is even higher (2), and those with IDD are often considered the largest global minority group (3). Unfortunately, health outcomes are consistently worse for those with IDD compared to those without IDD. Many decades worth of data consistently show higher rates of mental illness, addiction, obesity, in-patient care use, and all-cause mortality (4, 5) for those with IDD. Additionally, those with IDDs tend to experience increased social isolation (6), family stress (7), stigma (2), and education barriers (8). These challenges are likely even more extensive for children with IDD from underserved groups (e.g., rural and inner-city children, racial and ethnic minorities) (9–11). Though the needs of individuals with IDDs are often unique and complex, care teams serving those with IDD largely work in isolation from one another (e.g., education vs. healthcare). As a result, the public health burden due to IDD is high with few coordinated services that are often structurally disconnected by policy or funding siloes (12). For example, individuals with IDD and their families must navigate complex and fragmented networks of services, which limits providers' ability to gather a full understanding of the individuals they are serving.

Despite this high prevalence and public health burden, individuals with IDD still often struggle to live independently. Moreover, the approaches used to improve the lives of those with IDD are often designed to work within isolated disciples or for contained populations, which limits opportunities for innovation. Therefore, there is a tremendous need to support interdisciplinary work that develops novel approaches to define, measure, and intervene in order to improve outcomes for those living with IDD across the lifespan, and their families.

This collection focuses on enhancing the understanding of complex systems of care by creating a forum for interdisciplinary teams that explore innovative solutions to a variety of problems facing those with IDD. “Interdisciplinary” is broadly defined here and includes collaborative, family-centered approaches leveraging multiple systems. Collectively, these papers underscore a global commitment to advancing systems of care, professional training, and meaningful inclusion for individuals with IDD across the lifespan and address a wide range of critical areas of investigation. For instance, Ramachandran et al. review barriers to transitioning from pediatric to adult care for people with IDD, including limited provider training, poor care coordination, and lack of self-advocacy support. These authors recommend structured transition programs, enhanced provider education, and system-level reforms to ensure continuity and equity of care.

Three articles offer global perspectives. First, Zhong and Wijesinghe examine caregiving experiences among parents of children with cerebral palsy in Southwest China. They note that caregiver burden is influenced by child functioning, social support, and education, and the authors advocate for stronger psychosocial and policy supports to improve caregiver well-being. Second, Bakuwa et al. investigate the feasibility of a caregiver-led training model for parents of children with cerebral palsy in rural Malawi. They found this approach to be promising but highlight social and role challenges while recommending context-sensitive peer-led models to address workforce gaps. Third, Ferreira-Vasques et al. report on Brazilian norms for the Griffiths Scales of Child Development, Third Edition (GSCD-III), highlighting the importance of culturally relevant developmental measures and provides normative data for clinical and research use in Brazil.

Three other articles address capacity building projects that offer innovative approaches to improving accessibility and workforce development. Specifically, Fisher et al. discuss how the Leadership Education in Neurodevelopmental and Related Disabilities (LEND) program improves trainee knowledge and skill of how to effectively include people with disabilities in their work. Weber et al. describes a large workforce capacity building project called Project SCOPE. It is a scalable ECHO-based training program to enhance providers build capacity to work with children impacted by prenatal opioid exposure leading to neonatal abstinence syndrome. This article describes increased knowledge of intervention techniques, and confidence in implementation, as well as how the approach leads to exponential improvement in workforce capacity. Finally, Dudley et al. explore experiences of individuals with IDD participating in research to better understand how to support inclusive IDD research practices. Participants valued inclusion but noted accessibility and communication challenges. The authors suggest that developing accessible materials, supporting individuals with IDD to be in co-researcher roles, and ensuring financial compensation promote genuine inclusion and will improve research.

Finally, systems-level insights from public health and pandemic response are addressed in the last two articles. Specifically, Canpolat et al. examined national trends in special needs reports for children over a five-year period, finding disparities in access to services based on gender, geography, and socioeconomic status. The authors advocate for stronger data systems and targeted policy interventions to reduce inequities. Gotto et al., on the other hand, analyzed experiences with COVID-19 testing among children with IDD to identify broader lessons for care delivery. They found that communication difficulties, sensory sensitivities, and lack of provider preparedness were barriers to COVID testing, whereas caregiver advocacy, flexible procedures, and staff training in behavioral supports facilitated testing. The authors argue that these lessons can be applied to support more inclusive and adaptable healthcare practices beyond COVID-19.

Overall, these articles represent a diverse range of topics and fields, address complex and persistent challenges, and provide unique insights into solutions that have broad applicability. Together, they promote practical and exciting models for collaboration, training, and inclusion that center individuals with IDD and their families as partners in research, policy, and service design. However, ongoing research is needed to further develop these, and other innovations, that address challenges facing those with IDD, along with provision of robust person and familycentered support programs, and clinical interventions from an interdisciplinary lens. As this field develops, the use of inclusive, person/family-centered and interdisciplinary approaches will find the best, most effective solutions, and will create exciting new paths to allow those with IDD to thrive in their own communities on their own terms.
